# Investigation of anticoagulant rodenticide resistance induced by *Vkorc1* mutations in rodents in Lebanon

**DOI:** 10.1038/s41598-022-26638-5

**Published:** 2022-12-28

**Authors:** Antoine Rached, Georges Abi Rizk, Ali Barka Mahamat, Graziella El Khoury, Jeanne El Hage, Elena Harran, Virginie Lattard

**Affiliations:** 1 USC 1233 RS2GP, VetAgro Sup, INRAe, Univ Lyon, 69280 Marcy l’Étoile, France; 2 Animal Production department, Faculty of Agricultural Engineering and Veterinary Medicine, Lebanese University, Beirut, Lebanon; 3 Cambridge, CB4 1NE UK; 4 Animal Health Laboratory, Lebanese Agricultural Research Institute, Beirut, Lebanon; 5 Department of Biomedical and Pharmaceutical Sciences, National Higher Institute of Science and Technology, Abeche, Chad

**Keywords:** Ecology, Genetics

## Abstract

Anticoagulant rodenticides (AR) remain the most effective chemical substances used to control rodents in order to limit their agricultural and public health damage in both rural and urban environments. The emergence of genetically based resistance to AR worldwide has threatened effective rodent control. This study gives a first overview of the distribution and frequency of single nucleotide polymorphism in the vitamin K epoxide reductase subcomponent 1 (*Vkorc1*) gene in rodents in Lebanon. In the *Mus* genus, we detected two missense mutations Leu128Ser and Tyr139Cys*,* that confer resistance to anticoagulant rodenticides in house mice and a new missense mutation Ala72Val in the *Mus macedonicus* species, not previously described. In the *Rattus* genus, we found one missense mutation Leu90Ile in the roof rat and one missense mutation Ser149Ile in the Norway rat. This is the first study to demonstrate potential resistance to AR in Lebanese rodents and therefore it provides data to pest control practitioners to choose the most suitable AR to control rodents in order to keep their efficacy.

## Introduction

Ubiquitous in terrestrial ecosystems, rodents constitute the largest group of mammals in the world with approximately 2277 species^1^. Despite their ecological importance in plant propagation and beneficial effect on biotic environment, rodents cause serious damage in agricultural and urban areas and threaten public health and hygiene^2^. Rodents, whether native or introduced, when in close interaction with humans, are effective invaders. Indeed, they devastate agricultural production over large areas causing substantial economic damage in cereals and grain crops fields (rice, wheat, sugarcane, sorghum, etc.) and in orchard and plantation crops in different parts of the world^3^. Several diseases of humans, domestic animals and other wildlife species are epidemiologically linked to rodent populations as they can carry or transmit through their feces or urine many pathogens including plague, leptospirosis, trichinosis, Lassa fever, salmonellosis to name a few^4,5^. Additionally, rodents consume and spoil stored commodities to which they can access and gnaw through electrical cables and wirings, increasing food security and safety concerns, respectively^6,7^. The economic losses are often difficult to quantify, but they are estimated to be several billions of US dollars annually worldwide^8^. The three most successful cosmopolitan commensal rodents, the roof rat *Rattus rattus,* the Norway or brown rat *Rattus norvegicus* and the house mouse *Mus musculus*, are mainly distributed and documented worldwide due to their close association with people and their activities^9^. Given these prominent risks surrounding rodent infestations, appropriate site- and situation—specific control measures have become of critical global importance to alleviate pest rodent problems in accordance with an integrated pest management (IPM) strategy^10^. Different rodent management methods have been used, including lethal approaches like chemical rodenticides and physical traps or non-lethal procedures as habitat alteration, reproductive inhibition, ultrasonic devices and introduction of biological agent^11,12^. Chemical control with toxic substances is the most practical and cost-effective tool to mitigate damage caused by rodent populations over wide areas. Anticoagulant rodenticides, introduced during the late 1940s, demonstrated impressive efficacy. Their delayed onset of adverse effect, together with the high palatability of baits eliminated the problem of neophobia and bait shyness of rodents encountered with their predecessors, the acute acting toxicants such as thallium sulphate, strychnine, red squill, arsenic and zinc phosphide^13^. Anticoagulant rodenticides (AR) referred to as Vitamin K Antagonists (VKA) are derivatives of two compounds, indane-1,3-dione and 4-hyroxycoumarin with the exception of one thiocoumarin. They enact their anticoagulant properties by blocking the activity of the endoplasmic enzyme Vitamin K epOxide Reductase (VKOR), which disrupts the recycling pathways of vitamin K thus restricting the bioavailability of vitamin K involved in the blood clotting process leading to spontaneous bleeding and eventual death of the rodents^14^. The widespread use of the First Generation Anticoagulant Rodenticides, FGAR (*e.g.* warfarin, coumatetralyl and chlorophacinone) requiring multiple feeding in small amounts to maintain a lethal dose in rodents has selected many resistant rodent strains^15^. As a consequence of growing reports of resistance worldwide, new compounds referred to as Second Generation Anticoagulant Rodenticides, SGAR (bromadiolone, brodifacoum, difenacoum, difethialone and flocoumafen) were developed displaying more efficacy and potency for eradicating rodent infestations^13^. Nonetheless, resistance has evolved from FGAR to some SGAR molecules. This raises concerns about genetic resistance to anticoagulant rodenticides and increases attention to observing, mapping the expansion of resistance geographically and identifying its potential effect on VKA efficacy^16^. Not always, the mutations identified explain the resilience of VKOR enzyme to AR. Indeed, there are several different mechanisms reported across the world including the pharmacokinetic mechanism of resistance, gender-linked resistance, behavioral resistance, introgressed hybridization, intrinsic resistance and finally microbiome resistance^17^. This article will have a particular emphasis on genetic resistance. Briefly, the Vitamin K epOxide Reductase Complex subunit 1 (*Vkorc1*) gene that codes for the VKOR enzyme contains 163 amino acids^18^. Single nucleotide polymorphism (SNPs) can alter the amino acid sequence resulting in specific amino acid nonsynonymous (ns) substitutions in the three VKORC1 protein-encoding exons. These mutations have been shown to confer resistance to anticoagulant rodenticides. Hence, the development of populations of rats and mice resistant to AR since this acquired resistance is inheritable and stable. The degrees of resistance to VKA depend on the SNP mutations and whether the rodent is homogeneous or heterogeneous for this mutation. Given their similar mechanism of action, cross–resistance phenomena to both FGAR and SGAR can be established reducing the sensitivity of rodents to a broad range of VKA compounds. Nowadays, several *Vkorc1* nsSNPs are increasingly described in both developing and developed countries. Although this approach is interesting to understand the resistance mechanisms and its consequences in the field, it suffers from poor investigation in many regions of the world. Indeed, most of the studies are reported from western-European countries^19–21^. According to the resistance maps of the Rodenticide Resistance Action Committee (RRAC)^16^ and as far as we know, there is no published evidence of resistance to VKA in rodents from Middle East regions except one mutation found in Turkey^22^. This scarcity is more likely due to the lack of studies of resistance in those countries more than its absence. This also applies to Lebanon. With its distinctive topography comprising agricultural, coastal, mountainous and forest areas and its Mediterranean climate, Lebanon, the second smallest country in the Middle East, is endowed with an extremely rich flora and a high faunal diversity^23^. At present, this ecosystem biodiversity is threatened by an uncontrolled urban sprawl and demographic growth. Unsustainable management practices and overexploitation of natural resources have led to an increase in pollution level and hence climate change^24^. These ecological disturbances can be expected to alter the structure and dynamics of animal populations notably rodents leading to an increased emergence and affluence of species that thrive under these conditions to the detriment of native species^25^. What we know about Lebanese rodents is largely based on national surveys investigating endemic and non-endemic mammals to assess its conservation status through various invasive and non-invasive techniques^26–36^. However, from a health perspective, first and foremost is the sewer rats infestation in Beirut with *Leptospira interrogans*^5,37^ which raises public health concerns. While there was an unreliable report on the absence of resistance in rodent pest populations in Lebanon^38^, to-date there has been no scientific exploration of this matter.

This current study begins by a molecular identification of the commensal rodent species present in both urban and rural households in Lebanon, emphasizing knowledge of their negative impact within these areas. Then we will examine their genetic profile of *Vkorc1* to evaluate resistance to anticoagulant rodenticides in order to provide, at the end, an update of relevant control measures.

## Results

### Data collection

According to the Lebanese Ministry of Public Health (2018), the anticoagulant toxins currently used by registered companies for rodent control in Lebanon with their percentage of import for the year 2018 were the FGAR coumarin [coumatetralyl (0.0375% active ingredient)—2.9%.], the indanediones [Diphacinone (0.005%)—14.3%, Chlorophacinone (0.005%)—5.7%] and the SGAR hydroxycoumarins [Bromadiolone (0.005%)—54.3%, Brodifacoum (0.005%)—22.8%]. Moreover, zinc phosphide (0.005%) was also used as rodenticide.

### Rodent species identification

**Figure 1:**
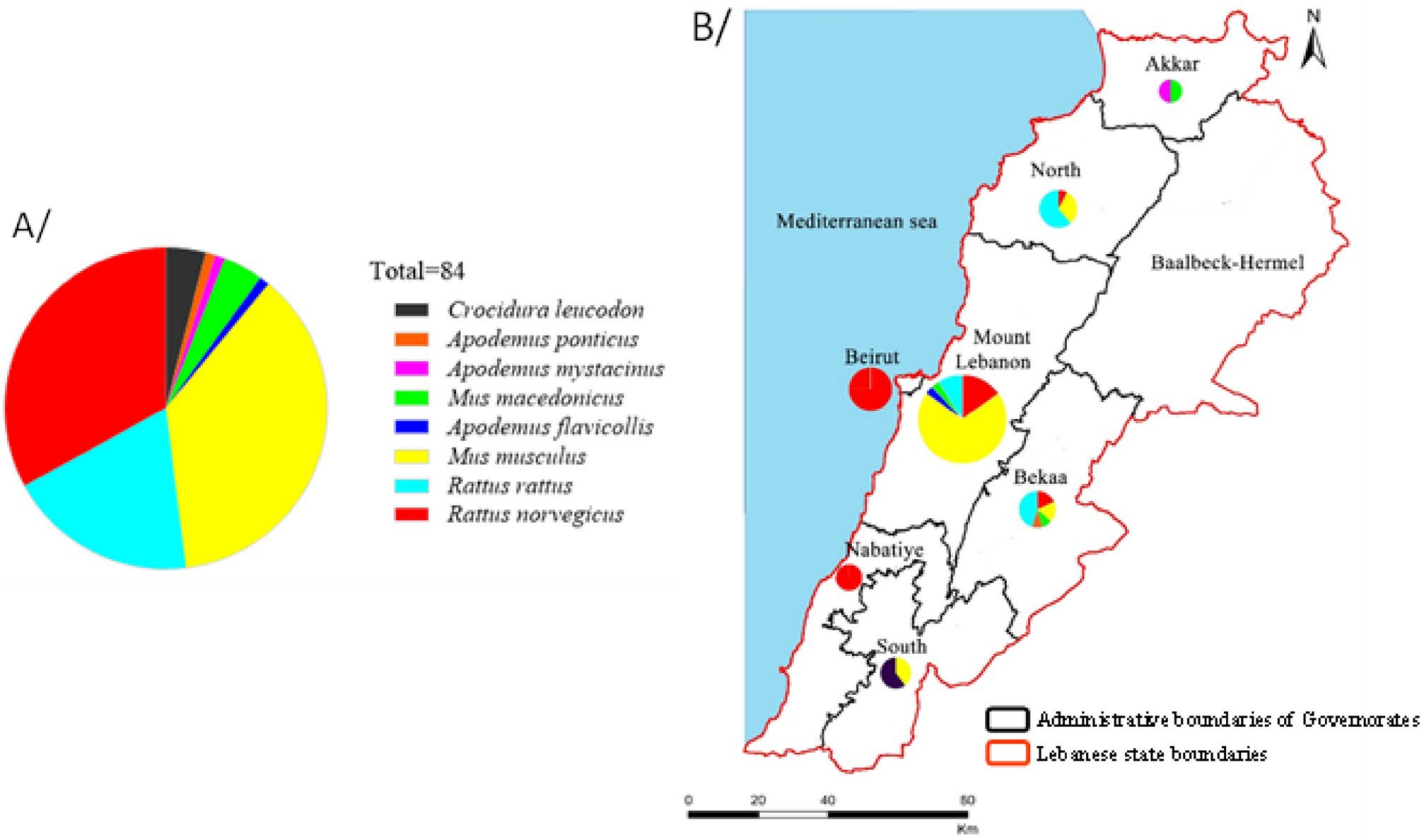
Distribution of species among (**A/**) Lebanon and (** B/**) the governorates of Lebanon.

In total, 92 animals were captured in this study. Of these, eight showed no PCR Cytochrome *b* amplification. In the remaining 84 field samples, eight species were identified, of which 81 (96%) belonged to the Muridae family. (31M*. musculus*, 28 *R. norvegicus*, 16 *R. rattus,* 3 *Mus macedonicus*, 1 *Apodemus mystacinus*, 1 *Apodemus ponticus* and 1 *Apodemus flavicollis*) and 3 (4%) belonging to the Soricidae family (*Crocidura leucodon*). An overview of PCR Cytochrome *b* specimen identifications and locality information are listed in Supplemental data S1 and indicated in Fig. 1. In all sites except Ouwaynat, at least one commensal species was captured. The presence of only one commensal species was recorded in 67% of the sites, while two commensal species was recorded in 28% of them.

Regarding the morphological features of the rodents, the Table 1 summarizes the external measurements. All the rodents captured had sharped head and round ears. However, fur color differed between species. The majority of R. rattus had black color and some individuals had grey–brown dorsal fur and a white belly. Of the study population of R. norvegicus, most of them were bicoloured, grey and black. Mus and Apodemus genus were plain-grey or brown-grey color. Crocidura leucodon had grey with shade of brown fur.

**Table 1:** Morphological measures* (cm) of the rodents captured.

Species	*R. rattus*	*R. norvegicus*	*M. musculus*	*A. flavicollis*	*M. macedonicus*	*C. leucodon*	*A. ponticus*
Head length	4.03 ± 0.76	4.05 ± 0.74	2.02 ± 0.46	2	1.73 ± 0.25	2.03 ± 0.06	2.5
Total (body and head length)	16.58 ± 2.48	16.61 ± 2.50	6.33 ± 1.06	5.8	5.83 ± 0.31	5.87 ± 0.06	7
Tail length	15.85 ± 2.09	15.86 ± 2.18	6.46 ± 0.81	6.2	5.93 ± 0.40	6.03 ± 0.15	7.5
Total	32.43 ± 4.26	32.46 ± 4.40	12.79 ± 1.66	12	11.77 ± 0.71	11.90 ± 0.1	14.5
Ear size	1.83 ± 0.37	1.86 ± 0.27	0.91 ± 0.25	1	0.80 ± 0.26	1	0.8

*Values represent the mean ± standard deviation (SD).

### *Vkorc1* genotyping of trapped rodent populations

Lebanese rats and mice were screened for mutations in the *Vkorc1* gene. Only *Vkorc1* sequences from 22 *R. norvegicus*, 6 *R. rattus*, 24 M*. musculus* and 2 *Mus macedonicus* were successfully exploitable. Details of mutations found in Lebanese rodents are presented in Table 2 and association of mutations found is listed in Supplemental data S2.

**Table 2:** Details of *Vkorc1* mutations found in Lebanese rodents.

Species	Mutation	Nucleotide position	Nucleotide WT	Nucleotide Mut	AA WT	AA mut	Exon
*Mus musculus*	Silent Mutation						
	E 37 E	111	A	G	Glu	Glu	1
	Missense Mutation						
	L 128 S	2190	T	C	Leu	Ser	3
	Y 139 C	2223	A	G	Tyr	Cys	3
*Rattus norvegicus*	Silent Mutation						
	H 68 H	1140	C	T	His	His	2
	I 82 I	1182	A	T	Ile	Ile	2
	Missense Mutation						
	S 149 I	2226	G	T	Ser	Ile	3
*Rattus rattus*	Silent Mutation						
	A 41 A	123	G	A	Ala	Ala	1
	I 82 I	1052	A	T	Ile	Ile	2
	L 94 L	1086	C	T	Leu	Leu	2
	I 107 I	1974	A	C	Ile	Ile	3
	T 137 T	2064	T	C	Thr	Thr	3
	A 143 A	2082	A	G	Ala	Ala	3
	Missense Mutation						
	L 90 I	1074	T	A	Leu	Ile	2
*Mus macedonicus*	Silent Mutation						
	E 37 E	111	A	G	Glu	Glu	1
	A 72 A	1010	G	A	Ala	Ala	2
	Missense Mutation						
	A 72 V	1009	C	T	Ala	Val	2
	Y 139 C	2223	A	G	Tyr	Cys	3

*WT* Wild-type sequence; *Mut* Mutated; *AA* Amino acid.

**Figure 2:**
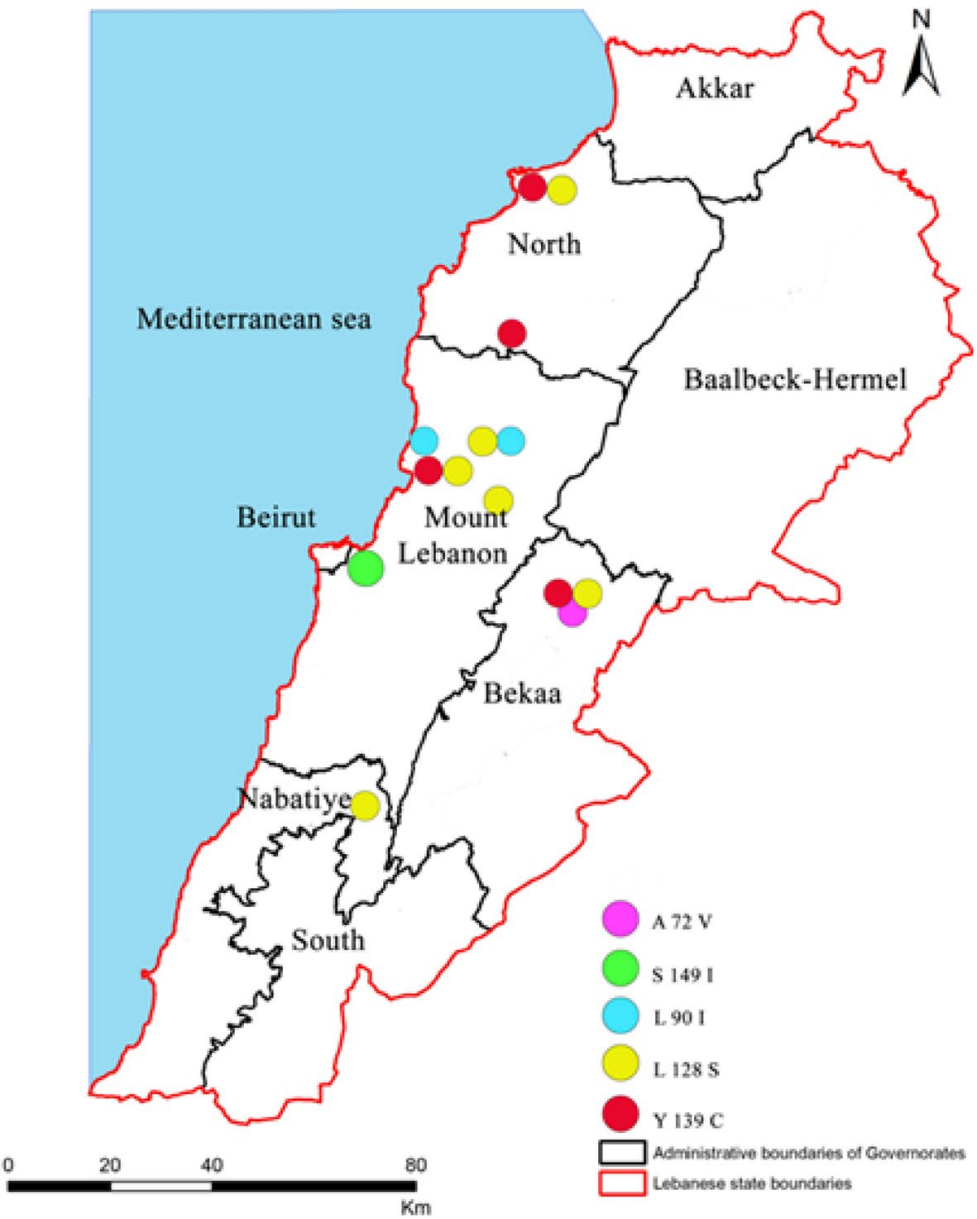
Distribution of *Vkorc1* mutations in the genus *Rattus* and *Mus* in the governorates of Lebanon.

In *R. norvegicus*, two associated silent mutations H68H and I82I (except for one sample in which H68H was alone) were detected in 91% of brown rats. A missense mutation S149I was detected in one sample in the heterozygous state in Mont Lebanon (Fig. 2). Its allelic frequency on the sampling was 2%.

In *R. rattus*, seven different mutations found either alone or in combination were detected, including six silent mutations distributed over the 3 exons A41A, I82I, L94L, I107I, T137T, A143A, and 1 missense mutation L90I found in two rats in the homozygous state in North Lebanon and Bekaa (Fig. 2). Allelic frequency of this mutation on the sampling was 33%.

Of the 24 M*. musculus* samples tested in this study, ten showed no sequence variation in the *Vkorc1* coding sequence gene (i.e., 41.6% of the sequenced samples from *M. musculus* species) and 14 presented missense mutations (i.e., 58.3% of the sequenced samples from *M. musculus* species). Three different mutations were detected. Two of these mutations were missense mutations L128S with an allelic frequency of 33% and Y139C with an allelic frequency of 8%. Six mice were heterozygous for the L128S mutation and five were homozygous. Two mice were heterozygous for the Y139C mutation and one was homozygous.

In *Mus macedonicus*, four different mutations were detected. Two of these mutations, E37E and A72A, were silent mutations and two, Y139C and A72V, were missense mutations, both found in the heterozygous state. Their respective allelic frequency was 25 for each of them.

## Discussion

Successful rodent control depends on the knowledge of the rodent species present to adapt the method to the behavior and ecology of the species in the environment where the problem arises. However, studies on rodent species present in the Middle East are often partial and/or old. To address this issue, a first step was to describe the species present in Lebanon, a Middle Eastern country with a wide range of climates due to its topographic diversity (mountainous/desert/Mediterranean regions), with both dense urban areas and agricultural areas, presenting numerous exchanges with Europe and other Middle Eastern countries. Considering the possible confusion of rodent species by a simple morphological approach, this identification was carried out by a dual morphological and genetic approach based on the sequencing of mitochondrial cytochrome b.

This study revealed in our sampling the presence of seven species belonging to the Muridae and one species belonging to the soricidae in urban and agricultural areas in Lebanon. The three commensal rodents *M. musculus*, *R. norvegicus* and *R. rattus* were predominant in our sampled sites with *M. musculus* seeming to be the most dominant species. These observations are coherent since these species are classified as invasive species that have colonized all continents except Antarctica and *M. musculus* and *R. rattus* are on the list of the 100 worst invasive alien species in the world. These three species were found to be common in livestock production units in Lebanon, certainly due to their availability in feed (fodder) and the protection provided to rodents against predators. This can result in serious economic losses and a significant public health threat because rodents can carry several pathogens that can be transmitted to livestock and people through their aerosols, feces, urine and ectoparasites^39–41^. In commercial, industrial and household sites, a dominance of *R. norvegicus* has been noted, with brown rats present in both urban and rural areas. This species was found to be widespread in human settlements and remarkably in Beirut which is consistent with the findings of Lewis et al.^26^. This may be attributed to the presence of sewage discharge^42^. In addition to the three commensal rodent species, our study describes a new rodent species in Lebanon *Apodemus ponticus* not previously recorded. It is also known as Black Sea field mouse. Its morphological features are close to those of the Lebanese *M. musculus* but smaller than those of the Iranian *Apodemus ponticus*^43^. The other species trapped in our study have already been described in Lebanon. According to Albaba^44^, *Crocidura leucodon* is found in Syria and Jerusalem. In our study, we trapped it only in Jezzine in southern Lebanon, which may indicate the possibility of its introduction from one of these two countries given the geographical proximity. *Apodemus mystacinus* found in the Ouwaynat-Akkar region has also been documented in the same region by Amr et al*.*^34^ and in a number of localities at different altitudes^26,33^. During 7 years of wild rodent collection, Lewis et al*.* failed to capture *Apodemus flavicollis* that was first found in Mount Hermon. However, in our study we captured one in Ghazir-Mount Lebanon supporting the hypothesis of its rare presence^26^. *Mus macedonicus* has been collected in three sites in Lebanon, including Akkar where it was also mentioned on the basis of cranial elements in the pellets of *Tyto Alba*^34^.

Commensal rodents are a great concern worldwide, they are involved in many types of damage, including agricultural and urban and are a public health risk. Anticoagulant rodenticides are the primary means of managing rodent populations among chemical pesticides across the world^45^. The surveys conducted in our study confirmed that rodent control in Lebanon also relies on the use of anticoagulant rodenticide in its two generic group FGAR and SGAR. This result raises the question of the quantity of anticoagulant rodenticides used in Lebanon. The only data present is that Lebanon imports about 6641 tons/year of all types of pesticides including rodenticides^46^ and the squirrel is close to extinction due to the excessive application of pesticides^31^. Moreover, there is no Lebanese governmental policies regarding the use of pesticides^50^. Thus, AR can be randomly used in Lebanon unlike other countries that have restricted the use of some AR compounds by the general public or amateurs in order to mitigate their hazards on human and non-target animals health and environment^47^. Therefore, rodent control in Lebanon is generally not performed by specialists. Unfortunately, an inappropriate and massive use of AR may further contribute to the selection of resistant individuals^48^, as largely described in the world^16,17,19–22^. The major mechanism supporting resistance to AR has been described to be due to mutations in the *Vkorc1* gene and different studies have been conducted in different countries to identify the mutations, characterize the degree of resistance conferred by mutations and determine their prevalence. The present study is the first to investigate *Vkorc1* polymorphisms in the Middle East, while resistance to AR has been reported worldwide and AR are routinely used.

In *R. norvegicus*, a substitution of serine amino acid with isoleucine (S149I) in one brown rat is reported here for the first time. The mutation had been previously observed in another species, i.e., *Arvicola terrestris* species in France. This mutation was shown to confer any resistance or limited resistance to AR^49^. Therefore, based on this result and even if this mutation is detected from a different species, we can speculate that this mutation is not associated to resistance to AR in *R. norvegicus*. Surprisingly, no other known missense mutations were detected in this study. This result is surprising compared to what has been described in many other countries. For example, in Europe, different mutations of *Vkorc1* in brown rats have been reported such as Y139F, Y139C, L120Q mutations associated with extreme resistance to FGAR. The prevalence of these mutations can reach up to 100% in some sites in Europe. Given the commercial exchanges between Europe and Lebanon, we would have expected the dispersion of these mutations in Lebanon. The use of AR by amateurs in Lebanon could be the cause of a significant use of SGAR unlike in European countries. This uncontrolled use of SGAR could possibly explain the non-dispersal of resistant rodents most likely coming from Europe by transport, as resistance to SGAR is still very sparsely described in Europe. Similarly, the alternate use of FGAR and SGAR has been proposed in China as one of the reasons why very little resistance exists in that country^50^. In Lebanese *R. norvegicus*, silent mutations have been detected. The silent mutation I82I has been detected in 89% of the *Vkorc1* sequences of rats of this study. It has been reported in *R. norvegicus* in England, Europe, Indonesia, Korea, the Azores and North and South America at a high frequency^21^. The H68H has already been described in China^50^. Although these point mutations do not alter the amino acid translation, Prescott et al*.* suggested that it could affect the efficiency of translation of the *Vkorc1* gene leading to an alteration of the biological response to anticoagulant exposure^51^. Further studies will be necessary to characterize the consequences of such silent mutations.

In *R. rattus,* the L90I substitution in the *Vkorc1* gene has been detected with an allelic frequency of 30% in Lebanon. This mutation has already been described throughout New Zealand with a frequency of 9%^52^. This mutation does not affect rats sensibility to AR since it conserves the same in vitro VKOR activity compared to wild type^21^. Compared to *R. norvegicus,* known *Vkorc1* mutations leading to severe resistance to AR are rare in *R. rattus* and it was proposed that the *Vkorc1*-based mechanism is not the major mechanism of resistance in this species.

In *M. musculus domesticus*, two already described mutations—i.e., Y139C and L128S—have been detected in house mice trapped in different Lebanese regions. Mice with L128S or Y139C substitutions in the *Vkorc1* gene have been found throughout United Kingdom (UK)^20^; European countries (Switzerland ; Azores^53^; Germany^21^; France^19^; Ireland^54^ and Serbia^55^) and United States of America^16^. These mutations are known to confer severe resistance to FGAR, bromadiolone and to a lesser extent to difenacoum^19^. Both mutations have been detected in house mice in the homozygous state demonstrating their high prevalence in Lebanon. The Y139C substitution was found in mice from North Lebanon and Bekaa, the L128S mutation in mice from North-, Mount—and South-Lebanon and Bekaa, suggesting a wide distribution of AR resistance in mice in Lebanon, as in the rest of the world. Yet, according to the Lebanese Ministry of Public Health (2018), more than 75% of the rodent control relies on the use of FGAR and bromadiolone. The use of such molecules cannot manage such resistant populations^19^. Therefore, control of these resistant mouse populations must rely on the use of other SGARs, which unfortunately are widely known to be associated with poisoning of non-target wildlife. Moreover, their over-intensive use could also lead to the emergence of new resistances by recombination between different genotypes and generation of populations carrying double mutations in the Vkorc1 gene, as has been observed in Europe^19^. Alternating solutions could be an interesting solution with rotation between ARs, but also with other non-anticoagulant solutions such as alphachoralose or cholecalciferol. These solutions have the advantage of being less ecotoxic for wildlife via ingestion of contaminated rodents because they are not persistent. On the other hand, they have been implicated in cases of primary poisoning of domestic wildlife through direct ingestion of baits and lack of antidote. Moreover, cholecalciferol may not be very effective in a warm country like Lebanon due to its mode of action.

The coding sequence of *Vkorc1* of only two *M. macedonicus* could be obtained in this study and the sequences of the 3 exons could be reported for the first time for this species. The consensus coding sequence is 99.6% identical to that of *M. musculus*. Two missense mutations with respect to the *M. musculus* sequence could be detected, both in the heterozygous state, each in a different individual. Surprisingly, one of the mutations is the Y139C mutation, also widely present in Lebanese *M. musculus*. As discussed above, this mutation confers severe resistance to AR. It is therefore demonstrated by this study that resistance to AR also exists in *Mus macedonicus*. Considering the low sampling of this species in our study, it is difficult to predict its prevalence. Nevertheless, the observation of this mutation in two individuals suggests a significant prevalence of this mutation in this species in Lebanon. The second non-coding mutation A72V, also detected in the heterozygous state, has never been described in *M. musculus* or other species. Therefore, its consequences for AR resistance are unknown. Nevertheless, the replacement of an alanine by a valine does not change the physicochemical properties or the steric hindrance of the residue, and should therefore not alter the properties of the corresponding VKORC1 protein.

This study focused on detecting *Vkorc1*-based resistance and demonstrates the presence of such mechanisms in Lebanon. Resistance mechanisms may be also due to other mechanisms, such as metabolic resistance, diet-based resistance^49^ and/or behavioral resistance^56–58^. Thus, in *R. rattus*, resistance could potentially be supported more by such mechanisms than by mutations in the *Vkorc1* gene. It would be important to conduct such a study in combination with an analysis of practical resistance in the field.

## Conclusion

In conclusion, the present study is the first to document the presence of *Vkorc1*-based resistance to FGAR and potentially to some SGAR in commensal rodents in the Middle East. While this resistance has so far only been demonstrated in *R. norvegicus*, *R. rattus* and *M. musculus*, our study also demonstrates the presence of such resistance in *M. macedonicus*. Surprisingly, the same Y139C mutation was found in *M. macedonicus* as in *M. musculus*. Is this a mutation or a recombination as described for the spretus genotype with an introgression of the *Vkorc1* gene from *Mus spretus* into the *M. musculus* genome^59^?

The results obtained in this study will guide future authorities in developing and implementing integrated management programs that combine efficiency and sustainability. Indeed, a good knowledge of the incidence and spread of rodenticide resistance is a prerequisite to select the most appropriate AR to preserve the effectiveness of anticoagulant rodenticides while addressing ecotoxicity issues.

## Methods

### Ethics statement

Rodent samples were collected from the national network of pest control operators. This study was thus not considered to be an “experimental procedure” as defined by the French and Lebanese legislation (Rural Code, Article R214–89) and was therefore not subject to an ethical committee approval. This study complied with the ethical standards of European regulations governing the care and use of animals in research (Directive 2010/63/EU) and it did not involve any endangered or protected species, or protected areas.

### Study area

Trapping campaign was performed throughout Lebanon during pest control campaign with the support of pest control operators, veterinary students and agronomists between February 2018 and May 2019. Rodents’ samples were collected by trapping with lethal traps, from 7 of 8 governorates throughout the territory to cover the main geographical zones. In each zone, different types of sites (agricultural, urban and households) were targeted.

**Figure 3:**
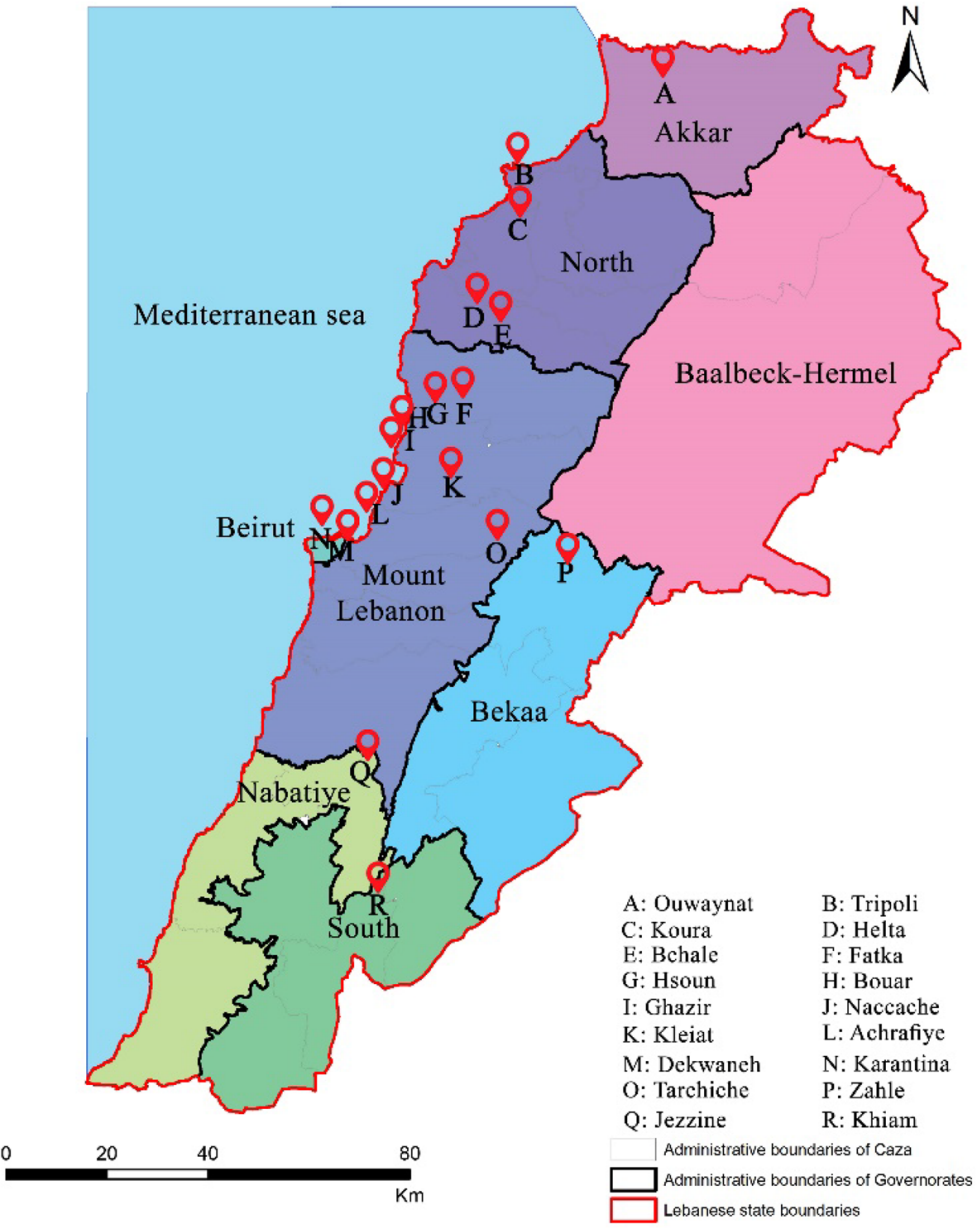
Trapping plots in Lebanon.

The distribution of successful capture sites in each governorate is shown in Fig. 3:

- North Lebanon : 4 sites (Tripoli, Koura, Helta and Bchale)
- Mount Lebanon : 7 sites (Ghazir, Kleiat, Hsoun, Bouar, Naccache, Fatka and Dekwaneh)
- Beirut : 2 sites (Achrafiye and Karantina)
- South Lebanon : 1 site (Jezzine)
- Nabatiyeh : 1 site (Khiam)
- Bekaa : 2 sites (Zahle and Tarchiche)
- Akkar : 1 site (Ouwaynat).

Regarding the type of the sites, Bchale and Achrafiye were households’ sites. Dekwaneh and Karantina were industrial/commercial sites and the remaining were animal production sites except Bouar, Naccache and Fatka. The rodents corresponding to these sites were provided dead without specifying the type of their origin site.

Data on rodent control products registered in Lebanon were provided by the Ministry of Public Health.

### Rodent tissue sampling

The process of rodents’ collection started by setting up traps in each zone. For each site, the trapping time was systematically performed over six consecutive days. Depending on the site size, 10 to 20 trapping stations were set up and they were homogeneously and consistently distributed over the area of interest (farms corners, sewage surroundings, wall cavities and food storage spaces). Lethal traps were coupled to strong-smelling baits with peanut butter, oat flakes and sardine oil. All traps were inspected daily and re-baited, if necessary.

Field specimen identifications (rat or mouse) were made, based on morphological criteria measured and recorded according to the reference^60^. Tail segment was cut of each dead rodent: 0.5 to 0.6 cm for rats and 1.1 to 1.2 cm for mice. The samples were sent to the laboratory in individual Eppendorf tubes in 70% ethanol solution. On receipt in the laboratory, samples were logged in and stored at − 20 °C pending analysis.

### Laboratory procedures

#### DNA extraction

Genomic DNA was extracted from tail sample with the GenElute™ Mammalian Genomic DNA Miniprep Kit (Sigma-Aldrich, Saint-Louis, Missouri, USA) following the manufacturer's instructions. The quality and yield of the Genomic DNA were determined spectrophotometrically using a nanodrop instrument.

#### Rodent species identification

We targeted the mitochondrial marker, the cytochrome *b* (Cy*t*.*b*) gene for species identification. The latter was amplified by Polymerase Chain Reaction (PCR). Primer sets used to amplify the Cyt.*b* are in Supplemental data S3^61^. The amplification was performed at 94 °C for 4 min followed by 40 cycles at 94 °C for 30 s, 50 °C for 30 s, 72 °C for 90 s, and a final 10 min at 72 °C^62^. PCR products were sent to Biofidal (Lyon, France) for sequencing.

The obtained sequences were compared to Cy*t*.*b* gene sequences in GenBank using the online nucleotide–nucleotide basic alignment program Blast search engine of the National Center for Biotechnology Information (NCBI). The search result was a listing of Cy*t*.*b* gene sequences corresponding to the rodent taxa, which are displayed in a descending degree of matching to the submitted sequence. To infer the rodent species, the highest percentage of nucleotide similarities was considered and genetic distance values lesser than 2% were indicative of intraspecific variation as suggested by Bradley and Baker^63^.

#### *Vkorc1* sequencing

Successful Cyt.*b* sequences were investigated for nucleotide sequence changes in the *Vkorc1* gene. Genomic DNA of the three exons of the rodent *Vkorc1* gene was amplified by PCR using specific primers of *Vkorc1* gene listed in Supplemental data S3. In order to sequence the totality of the *Vkorc1* gene, two sets of primers were used. Rat *Vkorc1* amplifications were carried out using rA-F and rA-R or rB-F and rB-R (10 pmol), while PCR reactions on the extracted mouse samples were performed with sA-F and sA-R or sB-F and sB-R (10 pmol). All amplifications were carried out in a final reaction volume of 25 μl containing 2 μl DNA, 2.5 μl 10X Accuprime buffer, Accuprime polymerase (1 unit, Invitrogen, Cergy Pontoise, France 12,346,086) and 200 μmol/L of each deoxynucleotide triphosphate. The amplification cycling conditions included one activation step at 94 °C for 3 min followed by 40 cycles of denaturation at 94 °C for 20 s, annealing at 62–64 °C for rat and mouse samples respectively for 20 s, elongation at 68 °C for 50 s for mouse samples and 2 min for rat samples, and a final extension step at 68 °C for 10 min. The amplified products were sequenced on both strands. The treated extracts were verified for the presence of amplicons on a 1% agarose gel by electrophoresis and quantified spectrophotometrically by a nanodrop instrument prior to sequencing. The *Vkorc1* exons 1, 2, and 3 from the rodent samples were sequenced by Biofidal (Lyon, France).

#### Genetic polymorphism analysis

Sequences obtained from mice, Norway rats and roof rats were inspected, edited and assembled using the software BioEdit software 7.2.5^64^. Subsequently, rat edited sequences were aligned in CLC Sequence Viewer version 7.6.1 software with the NCBI *R. norvegicus Vkorc1* (NM_203335.2) or *R. rattus* (LC_270886.1) *Vkorc1* reference sequences. The mice sequences were aligned using the NCBI *M. musculus Vkorc1* published sequence (NM_178600.2)^65^. The sequence trace files were visually compared with the reference sequences in order to reveal the homozygous mutations in the three exons. The heterozygous SNPs were confirmed by visual inspection of the sequencing electropherograms.

## Supplementary Information

Supplementary Information.
