# Supplementary material for: Investigation of anticoagulant rodenticide resistance induced by Vkorc1 mutations in rodents in Lebanon
Source: Sci Rep. 2022 Dec 28;12:22502. doi: 10.1038/s41598-022-26638-5 (PMC9797475; doi:10.1038/s41598-022-26638-5)
Supplement: Supplementary file 1 — Supplementary Information. [file 41598_2022_26638_MOESM1_ESM.docx]

| Governorate | Percentage of capture (%) | Sites | | Species |
| --- | --- | --- | --- | --- |
|  |  | number | identity |  |
| North Lebanon | 15.48 | 4 sites | Tripoli | *Rattus norvegicus* (1/4) |
|  |  |  |  | *Mus musculus* (3/4) |
|  |  |  | Koura | *Rattus rattus* (4/4) |
|  |  |  | Helta | *Rattus rattus* (4/4) |
|  |  |  | Bcheale | *Mus musculus (*1/1) |
| Mount Lebanon | 39.29 | 7 sites | Ghazir | *Rattus norvegicus* (2/20) |
|  |  |  |  | *Mus musculus* (16/20) |
|  |  |  |  | *Apodemus flavicollis* (1/20) |
|  |  |  |  | *Mus macedonicus* (1/20) |
|  |  |  | Kleiat | *Rattus norvegicus* (1/2) |
|  |  |  |  | *Mus musculus* (1/2) |
|  |  |  | Hsoun | *Mus musculus* (6/7) |
|  |  |  |  | *Rattus rattus* (1/7) |
|  |  |  | Bouar | *Rattus rattus* (1/1) |
|  |  |  | Naccache | *Rattus norvegicus* (1/1) |
|  |  |  | Fatka | *Rattus rattus* (1/1) |
|  |  |  | Dekwaneh | *Rattus norvegicus* (1/1) |
| Beirut | 15.48 | 2 sites | Achrafiye | *Rattus norvegicus* (6/6) |
|  |  |  | Karantina | *Rattus norvegicus* (7/7) |
| South Lebanon | 5.95 | 1 site | Jezzine | *Mus musculus* (2/5) |
|  |  |  |  | *Crocidura leucodon* (3/5) |
| Nabatiyeh | 8.33 | 1 site | Khiam | *Rattus norvegicus* (7/7) |
| Akkar | 2.38 | 1 site | Ouwaynat | *Mus macedonicus* (1/2) |
|  |  |  |  | *Apodemus mystacinus* (1/2) |
| Bekaa | 13.10 | 2 sites | Zahle | *Rattus norvegicus* (2/6) |
|  |  |  |  | *Mus musculus* (2/6) |
|  |  |  |  | *Mus macedonicus* (1/6) |
|  |  |  |  | *Apodemus ponticus* (1/6) |
|  |  |  | Tarchiche | *Rattus rattus* (5/5) |

**Supplemental data S1.** Distribution of PCR Cytochrome b - confirmed species among sites throughout Lebanon

| Geographic area | Species | Amino acid substitutions | Number of specimens | Silent Mutations |
| --- | --- | --- | --- | --- |
| North Lebanon | *Mus musculus* | Tyr139Cys | 2 | - |
|  |  | Leu128Ser | 1 | - |
|  | *Rattus rattus* | - | 1 | Ala41Ala |
|  |  | Leu90Ile | 1 | Leu94Leu, |
|  | *Rattus norvegicus* | - | 1 | His68His, Ile82Ile |
| Mount Lebanon | *Mus musculus* | Leu128Ser | 8 | - |
|  | *Rattus rattus* | - | 3 | Ala41Ala |
|  | *Rattus norvegicus* | - | 2 | His68His, Ile82Ile |
|  |  | - | 1 | His68His |
|  |  | Ser149Ile | 1 | His68His, Ile82Ile |
|  | *Mus macedonicus* | Tyr139Cys | 1 | - |
| Beirut | *Rattus norvegicus* | - | 10 | His68His, Ile82Ile |
| South Lebanon | *Mus musculus* | Leu128Ser | 1 | Glu37Glu |
| Nabatiyeh | *Rattus norvegicus* | - | 5 | His68His, Ile82Ile |
| Bekaa | *Mus musculus* | Tyr139Cys | 1 | - |
|  |  | Leu128Ser | 1 | - |
|  | *Rattus rattus* | Leu90Ile | 1 | Ile82Ile Leu94Leu, Ile107Ile, Thr137Thr, Ala143Ala |
|  | *Rattus norvegicus* | - | 1 | His68His, Ile82Ile |
|  | *Mus macedonicus* | - | 1 | Glu37Glu, Ala72Ala |
|  |  | Ala72Val |  | Glu37Glu |

**Supplemental data S2.** Association of silent and missense Vkorc1 mutations found in Lebanon

| **Gene Name** | | **Nucleotide sequence 5' → 3'** | **Fragment Length (base pairs)** |
| --- | --- | --- | --- |
| **Cytochrome *b*** | |  |  |
| Cyt.b-F | | ACC AAT GAC ATG AAA AAT CAT CGT T | 1213 |
| Cyt.b-R | | TCT CCA TTT CTG GTT TAC AAG AC |  |
| ***Vkorc1* Rat** | |  |  |
| Set A | rA-F | GGT TCT TCC CTC TTG TGT CTG | 1327 |
|  | rA-R | GGG TCA CCA AGA CAT GAG GTG |  |
| Set B | rB-F | AAG AGT AGG GGA CAA GGT GGC | 1354 |
|  | rB-R | ACT TGG GCA AGG CTC ATG TG |  |
| ***Vkorc1* Mouse** | |  |  |
| Set A | sA-F | GAT TCT TCC CTC CTG TCC | 1727 |
|  | sA-R | AGA CCC TGT CTC AAA ACC TA |  |
| Set B | sB-F | GAA AGC AGA ACA CTT AGC AGG | 1261 |
|  | sB-R | AAC CAA CAG CAG AAT GCA GCC |  |

**Supplemental data S3.** Primers used in this study
